# Supplementary material for: A UMLS-based spell checker for natural language processing in vaccine safety
Source: BMC Med Inform Decis Mak. 2007 Feb 12;7:3. doi: 10.1186/1472-6947-7-3 (PMC1805499; doi:10.1186/1472-6947-7-3)
Supplement: Additional file 13 — RAP application directory. Sets up directory for RAP (RDF application for PHP) source code [file 1472-6947-7-3-S13.gz › rap/doc/phpdoc/model/DbStore.html]

xml version="1.0" encoding="iso-8859-1"?


Docs For Class DbStore


## Class DbStore

Description

Description |
Methods (details)

DbStore is a persistent store of RDF data using relational database technology.

DbStore uses ADOdb Library for PHP V3.60 (http://php.weblogs.com/ADODB), which allows to connect to multiple databases in a portable manner. This class also provides methods for creating tables for MsAccess, MySQL, and MS SQL Server. If you want to use other databases, you will have to create tables by yourself according to the abstract database schema described in the API documentation.

History:

- access: public
- author: Radoslaw Oldakowski <radol@gmx.de>
- author: Daniel Westphal (http://www.d-westphal.de)
- version: V0.9.1

Located in /model/DbStore.php (line 32)

```
Object
   |
   --DbStore
```

Method Summary

Description |
Methods (details)

DbStore
DbStore
([string $dbDriver = ADODB\_DB\_DRIVER], [string $host = ADODB\_DB\_HOST], [string $dbName = ADODB\_DB\_NAME], [string $user = ADODB\_DB\_USER], [string $password = ADODB\_DB\_PASSWORD])

void
close
()

void
createTables
(string $databaseType)

boolean
datasetExists
($datasetName $datasetName)

object DatasetDb
getDatasetDb
($datasetId $datasetName)

object DbModel
getModel
(string $modelURI)

object NamedGraphMem
getNamedGraphDb
($modelURI $modelURI, $graphName $graphName)

object DatasetDB
&getNewDatasetDb
($datasetName $datasetName)

object DbModel
getNewModel
(string $modelURI, [string $baseURI = NULL])

object namedGraphDb
getNewNamedGraphDb
($modelURI $modelURI, $graphName $graphName, [$baseURI $baseURI = NULL])

void
isSetup
([string $databaseType = "MySQL"])

array
listModels
()

boolean
modelExists
(string $modelURI)

boolean
putModel
(object Model &$model, [string $modelURI = NULL])

boolean
removeNamedGraphDb
($modelURI $modelURI)

Methods

Description |
Methods (details)

Constructor DbStore (line 54)

Constructor: Set the database connection with the given parameters.

- access: public

DbStore

DbStore
([string $dbDriver = ADODB\_DB\_DRIVER], [string $host = ADODB\_DB\_HOST], [string $dbName = ADODB\_DB\_NAME], [string $user = ADODB\_DB\_USER], [string $password = ADODB\_DB\_PASSWORD])

- string
  $dbDriver
- string
  $host
- string
  $dbName
- string
  $user
- string
  $password

close (line 235)

Close the DbStore.

!!! Warning: If you close the DbStore all active instances of DbModel from this !!! DbStore will lose their database connection !!!

- access: public

void

close
()

createTables (line 87)

Create tables and indexes for the given database type.

Currently supported: MsAccess and MySQL. If you want to use other databases, you will have to create tables by yourself according to the abstract <a href="database\_schema.html">database schema</a> described in the API documentation.

- access: public
- throws: PhpError

void

createTables
(string $databaseType)

- string
  $databaseType

datasetExists (line 498)

Check if the Dataset with the given $datasetName is already stored in the database

- access: public
- throws: SqlError

boolean

datasetExists
($datasetName $datasetName)

- $datasetName
  $datasetName: string

getDatasetDb (line 520)

Create a new instance of DatasetDb with the given $datasetName and load the corresponding values from the database.

Return FALSE if the DbModel does not exist.

- access: public

object DatasetDb

getDatasetDb
($datasetId $datasetName)

- $datasetId
  $datasetName: string

getModel (line 161)

Create a new instance of DbModel with the given $modelURI and load the corresponding values of modelID and baseURI from the database.

Return FALSE if the DbModel does not exist.

- access: public

object DbModel

getModel
(string $modelURI)

- string
  $modelURI

getNamedGraphDb (line 543)

Create a new instance of namedGraphDb with the given $modelURI and graphName and load the corresponding values of modelID and baseURI from the database.

Return FALSE if the DbModel does not exist.

- access: public

object NamedGraphMem

getNamedGraphDb
($modelURI $modelURI, $graphName $graphName)

- $modelURI
  $modelURI: string
- $graphName
  $graphName: string

getNewDatasetDb (line 468)

Create a new instance of DatasetDb with the given $datasetName and insert the DatasetDb variables into the database.

Return FALSE if there is already a model with the given URI.

- access: public
- throws: SqlError

object DatasetDB

&getNewDatasetDb
($datasetName $datasetName)

- $datasetName
  $datasetName: string

getNewModel (line 185)

Create a new instance of DbModel with the given $modelURI and insert the DbModel variables into the database.

Return FALSE if there is already a model with the given URI.

- access: public
- throws: SqlError

object DbModel

getNewModel
(string $modelURI, [string $baseURI = NULL])

- string
  $modelURI
- string
  $baseURI

getNewNamedGraphDb (line 570)

Create a new instance of namedGraphDb with the given $modelURI and graphName and insert the DbModel variables into the database (not the graphName. This is only stored persistently, when added to dataset).

Return FALSE if there is already a model with the given URI.

- access: public
- throws: SqlError

object namedGraphDb

getNewNamedGraphDb
($modelURI $modelURI, $graphName $graphName, [$baseURI $baseURI = NULL])

- $modelURI
  $modelURI: string
- $graphName
  $graphName: string
- $baseURI
  $baseURI: string

isSetup (line 381)

Checks if tables are setup for RAP

- access: public
- throws: SqlError

void

isSetup
([string $databaseType = "MySQL"])

- string
  $databaseType

listModels (line 110)

List all DbModels stored in the database.

- access: public
- throws: SqlError

array

listModels
()

modelExists (line 139)

Check if the DbModel with the given modelURI is already stored in the database

- access: public
- throws: SqlError

boolean

modelExists
(string $modelURI)

- string
  $modelURI

putModel (line 211)

Store a MemModel or another DbModel from a different DbStore in the database.

Return FALSE if there is already a model with modelURI matching the modelURI of the given model.

- access: public

boolean

putModel
(object Model &$model, [string $modelURI = NULL])

- object Model
  &$model
- string
  $modelURI

removeNamedGraphDb (line 595)

Removes the graph with all statements from the database.

Warning: A single namedGraph can be added to several datasets. So it'll be removed from all datasets.

- access: public
- throws: SqlError

boolean

removeNamedGraphDb
($modelURI $modelURI)

- $modelURI
  $modelURI: string

#### Inherited Methods


Inherited From Object

> Object::toString()

Documentation generated on Thu, 7 Jul 2005 13:41:25 +0200 by phpDocumentor 1.3.0RC3
